# Effect of the beta-3 adrenergic receptor Trp64Arg and uncoupling protein 1–3826 A > G genotypes on lipid and apolipoprotein levels in overweight/obese and non-obese Chinese subjects

**DOI:** 10.1186/s12944-015-0029-y

**Published:** 2015-04-20

**Authors:** Yihong Chen, Xiaosu Wang, Zheni Shen, Ping Fan, Rui Liu, Yu Liu, Rongmei Ren, Lei Ma, Huai Bai

**Affiliations:** Laboratory of Genetic Disease and Perinatal Medicine and Key Laboratory of Birth Defects and Related Diseases of Women and Children of Ministry of Education, West China Second University Hospital, Sichuan University, Chengdu, 610041 Sichuan PR China; Division of Peptides related with Human Disease, West China Hospital, Sichuan University, Chengdu, 610041 Sichuan People’s Republic of China; Department of Biochemistry and Molecular Biology, West China School of Preclinical and Forensic Medicine, Sichuan University, Chengdu, 610041 Sichuan PR China

**Keywords:** Overweight/obese, $\beta$3-AR, UCP1, Gene polymorphism, PCR-RFLP

## Abstract

**Background:**

The beta-3 adrenergic receptor (*β3-AR*) Trp64Arg and uncoupling protein 1 (*UCP1*) -3826 A > G polymorphisms have been reported to be associated with obesity and/or lipid metabolism in some populations. This study examined the possible association of the *β3-AR* and *UCP1* polymorphisms with overweight/obesity or lipid variation in a Southwest Chinese population.

**Methods:**

A total of 418 Han Chinese (249 overweight/obese and 169 healthy control subjects) in the Chengdu area were studied using PCR-RFLP analysis. Total serum cholesterol (TC) and triglycerides (TGs) were measured using an enzymatic method. High density lipoprotein cholesterol (HDL-C) was determined after sodium phosphotungstate/magnesium chloride precipitation of low-density lipoproteins by polyvinyl sulfate. Serum apolipoproteins were quantified by radial immunodiffusion.

**Results:**

The genotype and allele frequencies of the *β3-AR* Trp64Arg and *UCP1* -3826 A > G polymorphisms in overweight/obese subjects exhibited no significant differences compared to the controls. However, subjects carrying the *β3-AR* TrpTrp genotype and *UCP1* AG genotype had higher TG levels than those carrying the Arg allele and AA genotype, respectively (P < 0.05), while controls carrying the *β*3*-AR* Arg allele had significantly higher TC and apo AII concentrations than those carrying the TrpTrp genotype (P < 0.05). Additionally, subjects carrying the *UCP1* AG genotype exhibited elevated apo C-II and apo C-III levels compared to those carrying the AA genotype (P < 0.05). We were unable to find an association of the *UCP1* and *β3-AR* polymorphisms with low HDL-cholesterolemia in the overweight/obese subjects.

**Conclusions:**

The present study provides evidence that the *β3-AR* Trp64Arg and *UCP1* -3826 A > G polymorphisms are associated with TG levels in overweight/obese Chinese subjects and that the two polymorphisms are also associated with certain lipid and apolipoprotein variations, depending on BMI. However, these polymorphisms are not associated with overweight/obesity or low HDL-cholesterolemia in a Chinese population from the Chengdu area.

## Background

Obesity is strongly associated with an adverse dyslipidemic profile and increased risks for diabetes, hypertension, and coronary artery disease. Although this relationship has been described in detail, its pathophysiological basis remains unclear.

Studies have suggested that obesity is linked to genetic and environmental factors and that most obesity-predisposing genes encode the molecular components of the physiological systems regulating energy balance [1].

The *β3-AR* gene has been established as the principal mediator of thermogenesis in brown adipose tissue and lipolysis in white adipose tissue both in animals and humans [2-4]. A *β3-AR* Trp64Arg polymorphism decreases receptor sensitivity [5,6] and may be associated with obesity and related traits [5,7,8]. The human uncoupling protein-1, which is expressed in brown adipose tissue, dissipates the transmitochondrial proton gradient as heat and plays an important role in energy homeostasis and thermogenesis [9,10]. *UCP1* gene polymorphisms have been implicated in the pathogenesis of obesity and related metabolic disorders, including lipid disorders [11,12]. A −3826 A > G polymorphism within the promoter region of the UCP-1 gene is a candidate gene polymorphic site related to these disorders [13-15]. Additionally, several studies have also suggested that the *UCP1* -3826 A > G and *β3-AR* Trp64Arg polymorphisms are related to low HDL-cholesterolemia and HDL-cholesterol levels in some populations [16-20]. However, the results of these studies were not confirmed in other studies [21-26]. Furthermore, no data concerning the relationships between the *β3-AR* Trp64Arg and *UCP1* -3826A > G polymorphisms and overweight/obesity and lipid profiles have been reported for a Southwest Chinese population. Therefore, we undertook this population-based study to assess whether the *β3-AR* Trp64Arg and *UCP1* -3826 A > G polymorphisms were related to overweight/obesity and lipid profiles in a Southwest Chinese population.

## Results

### Baseline characteristics of the participants in the population

The lipid and lipoprotein profiles for both the overweight/obese and control groups are provided in Table 1. The serum TG, apo CII, apo C-III and apoE levels were significantly higher and the HDL-C was significantly lower in the overweight/obese group compared to the control group (P < 0.05 or P < 0.01).

**Table 1:** **Clinical and biochemical characteristics of the overweight/obese and control subjects (** $\overline{\mathbf{x}}\pm \mathbf{s}$ **)**

	**Overweight/obese (n = 249)**	**Controls (n = 169)**	***P***
Age (yrs)	55.04 ± 10.00	53.35 ± 11.78	0.115
Gender (M/F)	149/100	93/76	0.322
TG (mmol/L)	2.31 ± 1.70	1.72 ± 1.56	0.000
TC (mmol/L)	5.43 ± 0.92	5.41 ± 1.11	0.788
HDL-C (mmol/L)	1.17 ± 0.32	1.41 ± 0.41	0.000
LDL-C (mmol/L)	^a^3.29 ± 0.87	^b^3.26 ± 1.04	0.821
apoA-I (mg/dL)	124.20 ± 27.96	128.97 ± 23.89	0.077
apoA-II (mg/dL)	28.39 ± 4.58	29.07 ± 6.92	0.227
apoB100 (mg/dL)	88.93 ± 17.06	84.04 ± 20.93	0.427
apoC-II (mg/dL)	6.68 ± 3.32	5.61 ± 3.10	0.001
apoC-III (mg/dL)	17.47 ± 7.03	13.09 ± 6.17	0.000
Apo E (mg/dL)	5.27 ± 1.96	4.87 ± 2.26	0.043
BMI (kg/m^2^)	25.72 ± 2.25	20.74 ± 1.60	0.000

Subjects with TG > 4.516 mmol/L (400 mg/dL) were excluded when calculating the serum LDL-C levels, ^a^: n = 193, ^b^: n = 145.

### Allele frequencies

The PCR amplified fragments (containing the $\beta$*3-AR* Trp64Arg and *UCP1* -3826 A > G polymorphic sites) from each sample were digested with the restriction enzyme Bst NI for the Trp64Arg site and Bcl I for the −3826 A > G site; then, the digested fragments were analysed by agarose gel electrophoresis.

Genotypes of the Trp64Arg and −3826 A > G polymorphisms were found to be in Hardy-Weinberg equilibrium in both the overweight/obese and control groups. The frequency data are presented in Table 2.

**Table 2:** **Genotype and allele frequencies of the** *β* ***3-AR*** **Thr64Arg and** ***UCP1*** **-3826 A > G polymorphisms in the overweight/obese and control groups**

	**Frequencies**		
	**Overweight/Obese (n = 249)**	**Controls (n = 169)**	***P***
*β3-AR* Trp64Arg Genotype			0.414
Trp Trp	0.739 (184)	0.734 (124)	
Trp Arg	0.253 (63)	0.243 (41)	
Arg Arg	0.008 (2)	0.024 (4)	
Allele			0.668
Trp	0.865 (431)	0.855 (289)	
Arg	0.135 (67)	0.145 (49)	
*UCP1* -3826 A/G Genotype			0.824
AA	0.249 (62)	0.225 (38)	
AG	0.454 (113)	0.479 (81)	
GG	0.297 (74)	0.296 (50)	
Allele			0.746
A	0.476 (237)	0.464 (157)	
G	0.524 (261)	0.536 (181)	

Numbers in parentheses indicate the number of subjects with each genotype or number of alleles of each type.

The Trp and Arg allele frequencies of the *β3-AR* gene at codon 64 in the overweight/obese and normal control groups were 0.865, 0.135 and 0.855, 0.145, respectively, while the A and G allele frequencies at the −3826 A > G site of the *UCP1* gene were 0.476, 0.524 and 0.464, 0.536, respectively. The allele frequencies of the two polymorphisms in the overweight/obese subjects were not different from those in the controls (P > 0.05).

Moreover, no significant difference in genotype frequencies was observed when the genotypes were divided into two categories according to the dominant model or recessive model at the *β3-AR* Trp64Arg and *UCP1* -3826 A > G sites (all P > 0.05). Multiple logistic regression analysis was used to assess the association of the *β3-AR* Trp64Arg and *UCP1* -3826 A > G polymorphisms with overweight/obesity. Assuming a dominant or recessive inheritance model, no association of the two polymorphisms were found after adjusting for age and sex (all P > 0.05).

### Effects of polymorphic sites of the *β3-AR* and *UCP1* genes on serum concentrations of lipids and apolipoproteins

To assess the possible impact of the polymorphic sites in the *β3-AR* and *UCP1* genes on lipid metabolism, we analysed serum lipid and lipoprotein levels in subjects possessing the different genotypes of the Trp64Arg and −3826 A > G polymorphisms in both the overweight/obese and normal control groups.

In the overweight/obese group, subjects with the TrpTrp genotype at the Trp64Arg site of the *β3-AR* gene had higher serum TG concentrations than the Arg allele carriers (TG: 2.40 ± 1.79 mmol/L vs 1.95 ± 1.11 mmol/L, P < 0.05) (Table 3), while in the normal controls the Arg allele carriers had higher TC and apoA-II concentrations than the TrpTrp genotype carriers (all P < 0.05) (Table 3).

**Table 3:** **Mean values** $\left(\overline{\mathbf{x}}\pm \mathbf{s}\right)$ **of serum lipid and apolipoprotein levels for the Thr64 Arg site of the** ***β3-AR*** **gene in the overweight/obese and control groups** ^**∆**^

	**Overweight/obese**		**Controls**	
	**TrpTrp (n = 184)**	**Trp Arg + ArgArg (n = 65)**	**TrpTrp (n = 124)**	**TrpArg + ArgArg (n = 45)**
TG (mmol/L)	2.40 ± 1.79	1.95 ± 1.11^a,*^	1.64 ± 1.05	2.05 ± 1.54
TC (mmol/L)	5.58 ± 1.02	5.33 ± 0.77	5.36 ± 1.04	5.77 ± 1.42^b,*^
HDL-C (mmol/L)	1.16 ± 0.31	1.24 ± 0.36	1.41 ± 0.41	1.44 ± 0.42
LDL-C (mmol/L)	^d^3.32 ± 0.91	^e^3.19 ± 0.73	^f^3.21 ± 1.01	^g^3.42 ± 1.15
apoA-I (mg/dL)	125.21 ± 29.49	124.33 ± 21.42	130.21 ± 22.58	126.13 ± 27.23
apoA-II (mg/dL)	28.54 ± 4.75	28.48 ± 4.90	28.53 ± 4.21	31.26 ± 11.61^c,*^
apoB100 (mg/dL)	93.44 ± 20.74	86.50 ± 14.41	89.38 ± 21.27	83.70 ± 23.98
apoC-II (mg/dL)	6.88 ± 3.50	6.12 ± 2.64	5.52 ± 2.95	5.99 ± 3.69
apoC-III (mg/dL)	15.92 ± 7.53	14.21 ± 4.79	13.04 ± 5.69	13.76 ± 7.67
Apo E (mg/dL)	5.39 ± 2.11	4.95 ± 1.42	4.75 ± 1.78	5.35 ± 3.29

^abc^Compared with genotype TrpTrp carriers in the same group, respectively, *P < 0.05.

Subjects with TG > 4.516 mmol/L (400 mg/dL) were excluded when calculating the serum LDL-C levels, ^d^: n = 142, ^e^: n = 51, ^f^: n = 108, ^g^: n = 37.

^∆^Age and sex were used as covariates in the analysis to adjust for the genotype effects.

In the overweight/obese group, subjects with the AG genotype of the A > G polymorphism of the *UCP1* gene had a higher serum mean concentration of TG, apo C-II and apo C-III compared to patients with the AA genotype (Table 4; all P < 0.05). No significant effect on lipid and apolipoprotein levels was observed for the *UCP1* -3826 A > G polymorphism in the normal control subjects.

**Table 4:** **Mean values (** $\overline{\mathbf{x}}\pm \mathbf{s}$ **) of serum lipid and apolipoprotein levels for the −3826 A > G site of the** ***UCP1*** **gene in the overweight/obese and control groups** ^**∆**^

	**Overweight/Obese**			**Controls**		
	**AA (n = 62)**	**AG (n = 119)**	**GG (n = 74)**	**AA (n = 38)**	**AG (n = 83)**	**GG (n = 53)**
TG (mmol/L)	1.92 ± 1.21	2.53 ± 1.94^a,*^	2.23 ± 1.50	1.58 ± 0.83	1.64 ± 1.19	1.88 ± 2.25
TC (mmol/L)	5.28 ± 1.00	5.43 ± 0.86	5.58 ± 0.90	5.39 ± 1.17	5.41 ± 1.03	5.38 ± 1.19
HDL-C (mmol/L)	1.20 ± 0.29	1.17 ± 0.33	1.17 ± 0.31	1.44 ± 0.51	1.43 ± 2.34	1.38 ± 0.41
LDL-C (mmol/L)	^d^3.16 ± 0.97	^e^3.24 ± 0.81	^f^3.44 ± 0.87	^g^3.20 ± 1.21	^h^3.21 ± 1.01	^i^3.19 ± 0.98
apoA-I (mg/dL)	126.06 ± 21.68	125.33 ± 34.42	120.59 ± 18.45	125.77 ± 25.14	130.36 ± 23.34	128.83 ± 24.14
apoA-II (mg/dL)	28.89 ± 4.49	28.38 ± 4.79	27.98 ± 4.35	28.52 ± 4.62	28.87 ± 8.82	29.71 ± 4.12
apoB100 (mg/dL)	100.15 ± 113.51	88.99 ± 17.13	91.14 ± 16.67	88.08 ± 25.74	91.23 ± 88.77	81.35 ± 19.56
ApoC-II (mg/dL)	5.81 ± 2.63	7.22 ± 3.66^b,*^	6.58 ± 2.91	5.31 ± 2.91	5.61 ± 2.81	5.73 ± 3.59
ApoC-III (mg/dL)	13.99 ± 6.75	16.30 ± 7.25^c,*^	15.24 ± 6.29	12.53 ± 5.23	13.04 ± 5.60	13.22 ± 7.52
Apo E (mg/dL)	4.93 ± 1.77	5.47 ± 2.07	5.18 ± 1.80	4.55 ± 1.54	4.89 ± 1.89	4.95 ± 3.08

^abc^Compared with AA genotype carriers in the same group, respectively, *p < 0.05;

Subjects with TG > 4.516 mmol/L (400 mg/dL) were excluded when calculating the serum LDL-C levels, ^d^: n = 43, ^e^: n = 90, ^f^: n = 60, ^g^: n = 34, ^h^: n = 71, ^i^: n = 40.

^∆^Age and sex were used as covariates in the analysis to adjust for the genotype effects.

### Evaluation of the relationship between polymorphic sites in the *β3-AR* and *UCP1* genes and low HDL-cholesterolemia

Previous studies have suggested that the *UCP1* -3826 A > G polymorphism was related to low HDL-cholesterolemia and low HDL-cholesterol levels in some populations with obesity. Additionally, the *β3-AR* Trp64Arg polymorphism has been shown to be related to low HDL-cholesterol levels in some ethnic groups. Therefore, we analysed the relationship between polymorphic sites in the *β3-AR* and *UCP1* genes and low cholesterolemia in our population.

In the overweight/obese subjects, the allele frequencies of the two polymorphisms in subjects with HDL-C < 1.04 mmol/L were not different from those with HDL-C ≥ 1.04 mmol/L (Table 5, both P > 0.05).

**Table 5:** **Genotype and allele frequencies of the** *β* ***3-AR*** **Trp64Arg and** ***UCP1*** **-3826 A > G polymorphisms according to HDL-C levels in the overweight/obese subjects**

	**Frequencies**		
	**HDL-C < 1.04 (n = 101)**	**HDL-C ≥ 1.04 (n = 148)**	***P***
*β3-AR* Trp64Arg Genotype			0.893^a^
Trp Trp	0.762 (77)	0.743 (110)	
Trp Arg	0.228 (23)	0.250 (37)	
Arg Arg	0.010 (1)	0.007 (1)	
Allele			0.794
Trp	1.752 (177)	1.736 (257)	
Arg	0.248 (25)	0.264 (39)	
*UCP1* -3826 A > G Genotype			0.243^b^
AA	0.198 (20)	0.291 (43)	
AG	0.485 (49)	0.446 (66)	
GG	0.317 (32)	0.264 (39)	
Allele			0.110
A	0.881 (89)	1.027 (152)	
G	1.119 (113)	0.973 (144)	

Numbers in parentheses indicate the number of subjects with each genotype or number of alleles of each type.

^a^ArgArg + TrpArg vs. TrpTrp, *χ* ^2^ = 1.118, odds radio (ArgArg + TrpArg/TrpTrp) = 0.902, 95% confidence interval 0.501-1.625.

^b^GG vs. AA + AG, *χ* ^2^ = 0.837, odds radio (GG/AA + AG) =1.296, 95% confidence interval 0.743-2.261.

## Discussion

Our results in the Chinese cohort living in Southwest China showed that both the polymorphisms in the *β3-AR* and *UCP1* genes were associated with TG concentrations in overweight/obese subjects and that the two polymorphisms were also associated with some lipid and apolipoprotein levels (i.e., TC, apoAII, apo CII and apo CIII levels) depending on BMI. However, we did not find an association between the polymorphisms and overweight/obesity or low HDL-cholesterolemia in overweight/obese subjects. These results provide support for the notion that *β3-AR* and *UCP1* are involved in the pathophysiology of metabolic disorders.

A total of 423 SNPs in *β3-AR* and 460 SNPs in *UCP1* in both the coding and non-coding regions are available in the public database. Although to date the function of a substantial number of the SNPs in the genes has not been defined, those linked to protein coding substitutions and in the introns, 5’ and 3’ UTRs related to the regulation of gene expression are of potential functional relevance. Among these SNPs, the most widely studied are the *β3-AR* Trp64Arg and *UCP1* -3826 A > G due to their functional importance.

There have been many studies concerning the relationship between the Trp64Arg polymorphism of the *β3-AR* gene and obesity or related phenotypes; however, the results are inconsistent. Clement et al. [5] reported that Arg64 resulted in an increased tendency to accumulate weight in French subjects and that this polymorphism was also associated with obesity [8], difficulty in weight loss and a lower basal metabolic rate [27] in Japanese subjects. In contrast, Ghosh et al. [28] reported that the *β3-AR* polymorphism was not associated with obesity in a large Finnish sample population, and Shiwaku et al. also did not find an association with moderately overweight Japanese workers [29]. Our results are in line with the reports of Ghosh et al. and Shiwaku et al. The discrepancy among these studies may be caused by several factors, including environmental and genetic factors.

With respect to the effect of the *β3-AR* Trp64Arg polymorphism on lipids and apolipoproteins, Kim-Motoyama et al. [20] reported that the polymorphism was associated with obesity and decreased serum TG levels. Our results in overweight/obese Chinese are in line with the report. A previous report suggested that subjects with this mutation may be characterized by decreased lipolysis in visceral adipose tissues [20]. In the current study, we also found that the Arg allele carriers showed increased TC and apo AII concentrations compared to Trp allele carriers in the normal control subjects, which is in general consistent with the notion that the *β3-AR* polymorphism is associated with cholesterol metabolism, including a variation of HDL-C components such as apoA-II. We were unable to find a significant association between the polymorphism and HDL-C levels in our population, although reports have suggested that the Arg64 allele carriers of the *β3-AR* gene had decreased HDL-C levels in some populations [19,20].

There are several reports available concerning the effect of the *UCP1* -3826A > G polymorphism on obesity or plasma metabolic parameters. However, controversy remains because some reports do not support this role. For instance, Urhammer et al. [30] showed that the *UCP1* polymorphism was not associated with obesity in a Danish population, and Pihlajamaki et al. [31] reported that BMI and serum biochemistry were not significantly different based on *UCP1* genotype in Finnish subjects with family combined hyperlipidemia. Schaffler et al. [26] showed that the G allele of *UCP1* was not associated with obesity and metabolic parameters in a large sample of German subjects. The current study is supportive of the results from the Danish, Finnish and German populations. Interestingly, we found that TG, apoC-II and apoC-III levels in overweight/obese subjects were elevated in AG genotype carriers compared with AA genotype carriers, suggesting a link between impaired metabolic levels in overweight/obese subjects and this polymorphism.

Kotani et al. reported that the *UCP1* -3826A > G polymorphism was associated with low HDL-cholesterolemia in obese Japanese patients [32]. In the current study we are unable to find a similar effect in overweight/obese subjects (OR = 1.296, CI = 0.743-2.261, P = 0.360), suggesting that *UCP1* gene variation might not predispose overweight/obese Chinese subjects to the development of low HDL-cholesterolemia. Our result is in line with the reports in German Caucasians [26] and young Japanese males [33]. Interestingly, we found that other gene polymorphisms, such as *ABCA1* R219K, were associated with low HDL-cholesterolemia in our overweight/obese subjects (n = 206, OR = 2.243, CI = 1.232-4.083, P = 0.008) (data not shown), suggesting that genetic variant(s) other than *UCP1* might predispose Chinese populations to low HDL-cholesterolemia.

Although the patient and control subjects participating in this study were randomized at enrollment into the respective overweight/obese and control groups, both groups had similar socioeconomic statuses (i.e., salary and education) and background environmental factors (i.e., diet and place of residence), and we attempted to control for some potential confounding factors such as age and sex in the data analyses, there might still have been some degree of uncontrolled confounding factors (i.e., smoking and forms of exercise). Future studies that examine whether any of these confounding factors additionally acted as effect modifiers on the relationship between the genotype and disease or related phenotypes will provide a more complete picture of genetic variations and the linked disease.

Finally, we should note that the difference in findings might reflect true variability in the association among different populations or ethnic groups. The above reports suggest that the role of the *β3-AR* and *UCP-1* polymorphisms is complex. Obesity and related metabolic disorders may be determined by multiple factors, such as environmental and life style components, in addition to genetic components in which many candidates genes and their interactions may be involved.

## Conclusions

The present study provides evidence that the *β3-AR* Trp64Arg and *UCP1* -3826 A > G polymorphisms are associated with TG levels in overweight/obese Chinese subjects and that the two polymorphisms are also associated with certain lipid and apolipoprotein variations, depending on BMI. However, these polymorphisms are not associated with overweight/obesity or low HDL-cholesterolemia in a Chinese population from the Chengdu area.

## Methods

### Subjects

For this study, blood samples were collected from 418 volunteers (242 men, 176 women, aged 53.77 ± 11.29 years) who were taking part in a routine health examination at the three hospitals of Sichuan University and Sichuan Normal University in Chengdu, China. All of these subjects were current or retired staff members of the Universities and were apparently healthy and unrelated individuals. After a 12 to 14 hour overnight fast, the blood from each individual was collected and analysed for serum concentrations of lipids, lipoproteins and apolipoproteins. BMI was calculated from height and weight measurements using the formula: BMI = body weight/(height)^2^ in kg/m^2^. According to the World Health Organization guidelines for Asians, individuals with BMI ≥ 23 kg/m^2^ are classified as overweight and those with BMI ≥ 25 kg/m^2^ are classified as obese [34]. Low HDL-cholesterolemia was defined as HDL-C < 1.04 mmol/L [35]. For the case–control study, 249 overweight/obese subjects (149 men, 100 women, aged 55.04 ± 10.00 years) with a BMI ≥23 kg/m^2^ and 169 normal weight (BMI < 23) subjects (93 men, 76 women, aged 53.35 ± 11.78 years) were used as the patients and controls, respectively. All of the subjects were Han Chinese living in the Chengdu area. Subjects with internal implications, such as CHD, diabetes mellitus and hypertension, were excluded. All study participants provided their informed consent, and the study was approved by the institutional review board of the hospitals of Sichuan University and Sichuan Normal University.

### Quantitative analysis

Total serum cholesterol (TC) and triglycerides (TGs) were measured by enzymatic methods (kits, Zhong Sen Co., Beijing). High density lipoprotein- cholesterol (HDL-C) was determined after sodium phosphotungstate/magnesium chloride precipitation of low-density lipoprotein by polyvinyl sulfate. Serum apo A-I, apoA-II, apo B100, apo C-II, apo C-III and apo E were quantified using a radial immunodiffusion kit developed by our laboratory [36]. The serum LDL-C concentration was calculated according to the Friedewald equation [37].

### DNA extraction and genotyping

Genomic DNA was isolated from 500 *μ*l of peripheral blood according to the method of Erlich [38]. The PCRs were performed in a final volume of 25 *μ*l containing 10% 10× PCR buffer, 2 mM MgCl_2_, 0.2 mM dNTPs, 0.5 U Taq DNA polymerase (MBI Fermentas) and 0.5 *μ*M of each sense and antisense primer. We used 100 ng of DNA per PCR reaction. Genotypes for polymorphic *β3-AR* the Trp64Arg and *UCP1* -3826 A > G polymorphisms were determined as previously described [7,18].

### Statistical analysis

Comparisons of continuous variables between two or more groups were performed with an independent *t*-test or one-way ANOVA, respectively. Allele frequencies of the *beta3-AR* and *UCP1* gene polymorphisms were estimated by the gene counting method. Allele and genotype frequencies were compared between cases and controls by a chi-square test. Genotypes were also assessed according to the dominant and recessive genetic model. Each genetic model comprised two groups: the combined group of variant homozygotes and heterozygotes vs wild-type homozygotes for the dominant model; and variant homozygotes vs the combined group of wild-type homozygotes and heterozygotes for the recessive model. Multiple logistic regression analysis with a stepwise forward selection procedure was performed to calculate odds ratios (OR) with obesity as a dependent variable and genotype according to the above genetic models in the presence of confounders (age and sex) as independent variables. Differences were considered statistically significant at a p-value <0.05. The statistical analyses were performed using the Statistical Package for Social Sciences (SPSS 11.0 for Windows).

## Abbreviations

*β*3-AR: Beta-3 adrenergic receptor
UCP1: Uncoupling protein 1
TC: Total cholesterol
TG: Triglycerides
HDL-C: High density lipoprotein-cholesterol
LDL-C: Low density lipoprotein-cholesterol
apo: Apolipoprotein
PCR-RFLP: Polymerase chain reaction-restriction fragment length polymorphisms
OR: Odds ratio
BMI: Body mass index
